# The alternative vagal maneuver; converting atrioventricular nodal re-entrant tachycardia by a rectal thermometer

**DOI:** 10.1093/omcr/omad143

**Published:** 2024-01-27

**Authors:** Yuval Avidan, Amir Aker, Vsevolod Tabachnikov

**Affiliations:** Department of Cardiology, Lady Davis Carmel Medical Center, Haifa, Israel; Department of Cardiology, Lady Davis Carmel Medical Center, Haifa, Israel; Department of Cardiology, Lady Davis Carmel Medical Center, Haifa, Israel

## Abstract

Vagal maneuvers are techniques used to increase parasympathetic tone, particularly useful in the management of hemodynamically stable supraventricular tachycardias. If ineffective, adenosine can be attempted. We present a patient with atrioventricular nodal re-entrant tachycardia (AVNRT), who could not effectively perform Valsalva maneuvers and had contraindications for carotid massage and adenosine administration, that converted into sinus rhythm by using a rectal thermometer. This maneuver was reproduced on various occasions. We suggest that rectal vagal maneuver may provide an additional therapeutic modality for selected patients with AVNRT.

## INTRODUCTION

Vagal maneuvers increase parasympathetic tone mediated via the vagus nerve. Cardiac response is divided into two parts: first, a negative chronotropic effect due to slowing the impulse generation at the sinoatrial node. Second, a negative dromotropic effect by prolonging the refractory period at the atrioventriuclar (AV) node and thereby slowing the conduction to the ventricles. Common maneuvers include carotid sinus massage (CSM), standard Valsalva maneuver (SVM) and its modified version. Although rarely used in clinical settings, forceful coughing, ocular pressure and rectal manipulation may result in similar effects [1]. Herein we describe a case of AVNRT in which rectal examination aided conversion to sinus.

## CASE REPORT

A 54-year-old male presented to the emergency department with sudden onset of palpitations. They appeared at rest without prodromal symptoms and lasted several hours. The patient described shorter self-resolving episodes in the past. His cognitive status was impaired following a cerebrovascular accident (CVA) and he was confined to a wheelchair after bilateral below knee amputation as a complication of diabetes and peripheral vascular disease (PVD). Past history included drug abuse and chronic obstructive pulmonary disease (COPD).

**Figure 1:**
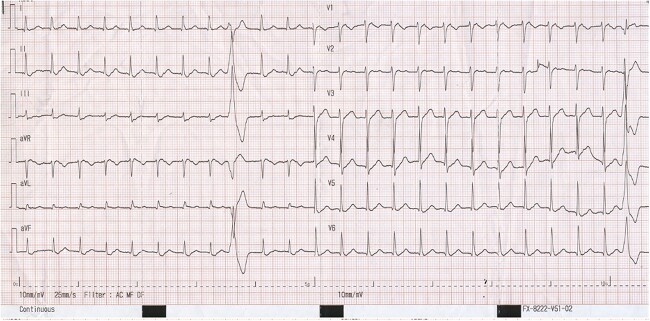
The ECG upon admission shows a regular narrow complex tachycardia at a rate of 145/bpm with a retrograde P wave visible between the QRS and T wave (best at lead V1), resulting in a short-RP interval (95 ms), suggestive of typical AVNRT. There are multiple monomorphic PVCs.

Vitals at presentation were within normal limits except for pulse rate of 145 bpm. Examination revealed no abnormalities aside from the tachycardia. Electrocardiogram (ECG, Fig. 1) showed a regular narrow complex tachycardia with retrograde P waves visible between the QRS and T wave (best at lead V1), resulting in a short-RP interval, measured at 95 milliseconds, suggestive of AVNRT of the typical form. There were multiple monomorphic premature ventricular contractions (PVCs).

Next, various VM were attempted. The patient was asked to take a deep breath and blow out against a closed glottis, which he was unable to perform effectively. He was then instructed to blow into the tip of a 10 ml syringe after forced expiration, once again ineffectively due to lack of cooperation. Taking his history into account, namely COPD, as well as the fact he repetitively declined the administration of medications, we decided to refrain from adenosine administration. CSM was contraindicated as there was a history of CVA.

**Figure 2:**
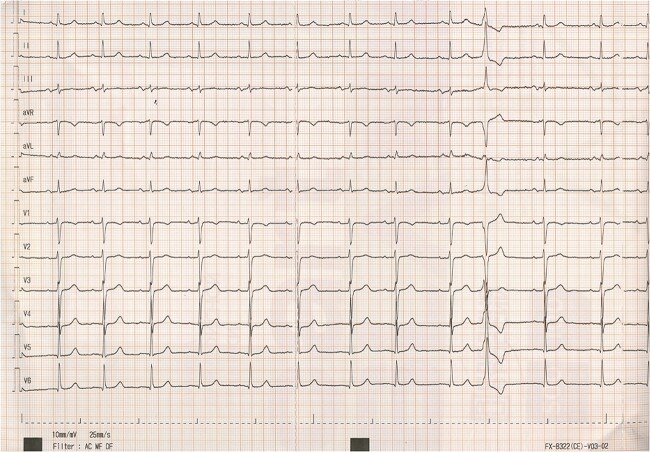
The ECG immediately after conversion into sinus rhythm. Note the same pattern of PVC, which has been described in literature as a potential trigger of AVNRT.

Simultaneously, the nursing staff obtained his core temperature using a rectal thermometer in order to verify the absence of fever. During the measurement he immediately converted into sinus rhythm (Fig. 2). No AV nodal blocking agents or antiarrhythmic drugs were given prior to the conversion. The patient was reluctant to continue medical therapy or to consider to undergo an ablation procedure.

Interestingly, a month later he was admitted with COPD exacerbation and had two additional episodes of narrow-complex tachycardia, terminated in a similar fashion. The patient was then lost to follow-up for several months.

## DISCUSSION

AVNRT is a type of paroxysmal supraventricular tachycardia (SVT) that results from a reentry circuit within or adjacent to the AV node. Dual AV node anatomy is pivotal in the genesis of AVNRT, forming two pathways that exhibit different refractoriness and conduction velocities, which enables impulse propagation and development of self-sustaining tachycardia. AVNRT is responsible for about 60% of all SVT cases. It is more prevalent in women and can present in all age groups, although it has a bimodal distribution over time [2, 3].

As reflected in current guidelines, VM and CSM are the preferred management of hemodynamically stable SVT. When performed correctly, their success rate may reach up to 54% [2]. Various studies suggested different rates of efficacy and safety of vagal maneuvers, these characteristics are summarized in Table 1, reproduced from Huang et al. [4]. As described in the case, there are various variations of VM, all require full cooperation from the patient. These are collectively referred to as standard Valsalva maneuver (SVM). The modified Valsalva maneuver (MVM), refers to a postural modification to the SVM and has been shown to be superior in the successful conversion of SVT to sinus rhythm when compared to other maneuvers [4].

**Table 1:** Different reported rates of efficacy and safety of various vagal maneuvers, reproduced from Huang et al. [4]

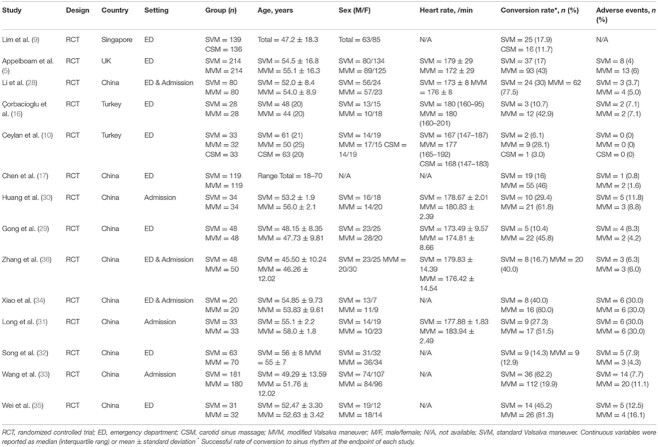

The CSM is performed by identifying the point of maximum carotid impulse and exerting pressure for a period of 10 seconds using longitudinal movements. Since CSM carries a risk of neurological complications, greater if there is preexisting carotid stenosis or carotid plaques, before attempting the maneuver the physician must verify the absence of a carotid bruit, history of transient ischemic attack or CVA. Adenosine administration in incremental dosing is recommended if these maneuvers are unsuccessful. However, as adenosine produces bronchoconstriction, it should be used cautiously in patients with COPD and asthma. In case these are ineffective, non-dihydropyridine calcium channel blockers and beta blocker should be considered, before proceeding with synchronized electrical *cardioversion* [2].

The rectum is innervated with nerve fibers that are sensitive to pressure. Rectal maneuvers may elicit increased parasympathetic tone via the stimulation of acetylcholine receptors located in the rectum [1]. In 1987, Roberge and colleagues reported a case when rectal maneuvers resulted in the termination of SVT [5]. In fact, besides the temporary inconvenience, rectal manipulation (e.g. digital rectal examination, temperature measurement) has obvious advantages. It is quick, reproducible and can easily be performed in patients with relative or absolute contraindications for the above-mentioned Vagal maneuvers. It should be avoided in neutropenic immunocompromised patients [6]. This method is probably underutilized because of the associated inconvenience. However, as suggested by this case, it may provide an additional therapeutic modality for selected patients with AVNRT.
